# Why every lab needs a handbook

**DOI:** 10.7554/eLife.88853

**Published:** 2023-07-03

**Authors:** Benjamin C Tendler, Maddie Welland, Karla L Miller, The WIN Handbook Team, Melanie Alexis-Butler, Aurea Martins Bach, Emma Černis, Mark Chiew, Stuart Clare, Madalena Fonseca, Amy Howard, Daniel Kor, Kaitlin Krebs, Clemence Ligneul, Caitlin O’Brien, Thomas Okell, Daniel Papp, Sophie Schauman, Mo Shahdloo, Yuriko Suzuki, Cristiana Tisca, Yan Tong, Wenchuan Wu

**Affiliations:** Nuffield Department of Clinical Neurosciences, University of OxfordOxfordUnited Kingdom; Wellcome Centre for Integrative Neuroimaging, FMRIB, Nuffield Department of Clinical Neurosciences, University of OxfordOxfordUnited Kingdom; Department of Psychiatry, University of OxfordOxfordUnited Kingdom; Wellcome Centre for Integrative Neuroimaging, FMRIB, Nuffield Department of Clinical Neurosciences, University of OxfordOxfordUnited Kingdom; Wellcome Centre for Integrative Neuroimaging, FMRIB, Nuffield Department of Clinical Neurosciences, University of OxfordOxfordUnited Kingdom; Wellcome Centre for Integrative Neuroimaging, FMRIB, Nuffield Department of Clinical Neurosciences, University of OxfordOxfordUnited Kingdom; Wellcome Centre for Integrative Neuroimaging, FMRIB, Nuffield Department of Clinical Neurosciences, University of OxfordOxfordUnited Kingdom; Wellcome Centre for Integrative Neuroimaging, FMRIB, Nuffield Department of Clinical Neurosciences, University of OxfordOxfordUnited Kingdom; Wellcome Centre for Integrative Neuroimaging, FMRIB, Nuffield Department of Clinical Neurosciences, University of OxfordOxfordUnited Kingdom; Wellcome Centre for Integrative Neuroimaging, FMRIB, Nuffield Department of Clinical Neurosciences, University of OxfordOxfordUnited Kingdom; Wellcome Centre for Integrative Neuroimaging, FMRIB, Nuffield Department of Clinical Neurosciences, University of OxfordOxfordUnited Kingdom; Wellcome Centre for Integrative Neuroimaging, FMRIB, Nuffield Department of Clinical Neurosciences, University of OxfordOxfordUnited Kingdom; Wellcome Centre for Integrative Neuroimaging, FMRIB, Nuffield Department of Clinical Neurosciences, University of OxfordOxfordUnited Kingdom; Wellcome Centre for Integrative Neuroimaging, FMRIB, Nuffield Department of Clinical Neurosciences, University of OxfordOxfordUnited Kingdom; Wellcome Centre for Integrative Neuroimaging, FMRIB, Nuffield Department of Clinical Neurosciences, University of OxfordOxfordUnited Kingdom; Wellcome Centre for Integrative Neuroimaging, FMRIB, Nuffield Department of Clinical Neurosciences, University of OxfordOxfordUnited Kingdom; Wellcome Centre for Integrative Neuroimaging, FMRIB, Nuffield Department of Clinical Neurosciences, University of OxfordOxfordUnited Kingdom; Wellcome Centre for Integrative Neuroimaging, FMRIB, Nuffield Department of Clinical Neurosciences, University of OxfordOxfordUnited Kingdom; Wellcome Centre for Integrative Neuroimaging, FMRIB, Nuffield Department of Clinical Neurosciences, University of OxfordOxfordUnited Kingdom; 1 Wellcome Centre for Integrative Neuroimaging, FMRIB, Nuffield Department of Clinical Neurosciences, University of Oxford Oxford United Kingdom

**Keywords:** research culture, lab handbooks, early-career researchers, principal investigators, careers in science, onboarding

## Abstract

A lab handbook is a flexible document that outlines the ethos of a research lab or group. A good handbook will outline the different roles within the lab, explain what is expected of all lab members, provide an overview of the culture the lab aims to create, and describe how the lab supports its members so that they can develop as researchers. Here we describe how we wrote a lab handbook for a large research group, and provide resources to help other labs write their own handbooks.

## Welcome to the lab

Imagine that two new early-career researchers are joining your lab. Everybody in the lab is friendly and supportive, and before long they are busy doing research! Although they have been given clear instructions about their research projects, nobody has explained to the recent arrivals how the group works on an interpersonal level, so they begin to form their own impressions.

One of the new lab members notices that everybody seems to be highly productive – publishing papers, managing experiments, and generally pushing back the frontiers of science. They conclude that the principal investigator (PI) must expect everyone to work long hours to make similar progress. One day, this researcher has a brilliant idea, and writes a long email to the PI describing it in great detail. After a few days, the PI sends a short reply, “let’s discuss later”, so the researcher concludes that the PI did not like the idea.

The other new arrival is more experienced, but is worried about an upcoming conference that they feel they have to attend because other members of the lab are going. This researcher does not feel comfortable travelling to the country where the conference will be held for personal reasons, but they are not sure if they should raise this with the PI as no one else has raised concerns.

It is not just early-career researchers who are affected by uncertainty. An experienced technician, for example, is frustrated because they were not included as an author on a paper to which they feel they made a substantial contribution. A PI notices that someone in the lab seems to be struggling to find motivation, but is unsure about the best way to start a conversation with them while being sensitive to both their privacy and wellbeing. More generally, the PI wants to know if members of the lab are happy with their working environment, but they do not know how to solicit sincere feedback.

These situations arise in part because there is no single rulebook for workplace culture in academic research. Many of the problems that happen in labs have their origins in a failure to clearly communicate the ethos of the lab, including what is expected of lab members with regard to collegial behaviour and interactions. For example, if the PI is not clear about their expectations in terms of working hours, a lab member may end up spending too much time in the lab (and run the risk of burnout; Woolston, 2021), or they may adopt a schedule that isn’t compatible with the way the rest of the lab works (which could lead to friction with the PI). It is also important to ensure that daily lab tasks and responsibilities are shared fairly, and that everyone has an opportunity to discuss their personal needs with the PI.

One way to minimise these challenges is to have a document, which we call a lab handbook, that describes the ethos of a lab, and explains how the group aims to create and maintain this ethos on a practical, day-to-day level. Resources that can be used to create a handbook for a lab are described in Box 1.

Box 1. **Resources for writing a lab handbook.**

To help other groups write their own lab handbook, we have established a set of publicly available resources (Tendler et al., 2022). These resources include (i) an abridged version of the lab handbook written by the Physics group at the Wellcome Centre for Integrative Neuroimaging (WIN); (ii) a video summarising the content of this article that can be used to initiate discussions with group members; (iii) a template containing a series of questions, the answers to which can be used to form the skeleton of a new handbook.

Other useful resources and example lab handbooks are available online, including handbooks produced by the Whitaker Lab at the Turing Institute (Whitaker Lab, 2021), and the Aly Lab at Columbia University (Aly Lab, 2022; Aly, 2018).

## Lab handbooks: Communicating culture

A good lab handbook will explain what is expected from lab members and the opportunities that are available to them; it will cover all career stages and all job roles; it will describe the culture and atmosphere that the lab aspires to; and it will articulate how the lab supports the professional development of its members. Crucially, a lab handbook is not like a technical manual that explains, for example, how to perform a certain experiment or to use computing resources (although such manuals are also valuable).

In addition to making sure that all lab members receive a consistent message and new starters feel welcome (Andreev et al., 2022), a good lab handbook will ensure the following:

- Lab members will have a pathway for accountability. Lab handbooks codify a social contract from which problematic behaviour can be identified and challenged. Handbooks should explicitly outline how to respectfully raise concerns when the behaviour of someone in the lab – including the PI – is not consistent with the expectations described in the handbook. It should also highlight institutional resources available to report more serious offenses, such as bullying and harassment.
- Lab members will feel empowered. The most effective lab handbooks are ‘living documents’ that evolve over time: if the handbook is reviewed on a regular basis, it will provide an opportunity for lab members to discuss how the group currently operates and to suggest changes.
- Lab members will understand how the lab functions. Being explicit about the roles of different group members and how they are expected to interact will help people communicate effectively and work together productively. It also provides an opportunity to emphasise the value of diverse roles and contributions.

Lab handbooks also help to promote wellbeing: writing down the expectations for a lab helps to reduce anxiety and avoid misunderstandings. Crucially, some lab members may be more comfortable reading about certain topics, such as mental health, than discussing them in person.

So far, we have focused on the benefits to most group members – what about PIs? It is possible that some PIs might be reluctant to take time away from research to create a lab handbook, so here is a list of potential benefits that can be used to convince PIs and other group members of the benefits from having one. First, it will enable them to deliver the leadership that their group members probably want. A recent survey of almost 4000 researchers found that only 41% of respondents thought that their “leaders communicate clear expectations regarding behaviours and/or culture” (Wellcome Trust, 2022), and an informal poll at our centre found unanimous support for handbooks from early-career researchers.

Second, articulating the ethos of a lab is constructive. It can be easy to allow practices and attitudes in the lab to evolve unchecked; however, if a research group has to articulate their vision for their lab in writing, it will force them to think more deliberately and strategically about how to create (and communicate) the culture they want to create and maintain.

Third, the act of writing can bring a lab together. If all lab members are involved in the creation of the handbook, it will create opportunities for discussions about topics that matter to them. Being empowered in this way can improve the job satisfaction and wellbeing of lab members.

Fourth, a lab handbook that lays out a supportive and positive culture can aid recruitment if some or all of it is made public. While we would discourage groups from undertaking a lab handbook for cynical reasons, co-creating a lab handbook can also be a great way to demonstrate a commitment to positive research culture where funding bodies expect this.

We strongly recommend that a lab writes its handbook collaboratively as a group, alongside the PI, rather than leaving it to one person to do all the writing. In addition to sharing the workload, writing as a group helps to achieve buy-in and fosters discussion. Importantly, handbooks do not need to be long to be useful: a short handbook that has been completed is better than a long handbook that has not.

## Case study: The WIN Physics lab handbook

**Figure 1.**
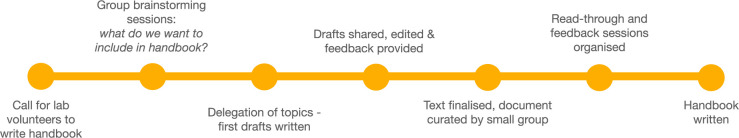
The process for writing the WIN Physics lab handbook. A lab handbook brings many benefits to a lab. Writing a lab handbook as a collaborative group exercise helps to achieve buy-in and encourages discussion.

The Physics group at the Wellcome Centre for Integrative Neuroimaging (WIN) has around 30 members. At the time we wrote our lab handbook, the group consisted of 4 PIs (including KLM), 5 members of core staff, around 10 early-career researchers (including BCT), and around 10 doctoral students. The approach we took to writing the handbook is outlined in Figure 1. A group of about 15 volunteers within the group held a series of brainstorming sessions to decide what our handbook would contain. We also read example handbooks from other labs to help us select high-level topics for our own handbook, initially guided by a comprehensive Twitter thread from Sam Mehr of the University of Auckland (Mehr, 2019). Once the high-level topics had been finalised, responsibility for drafting the relevant sections of the handbook was delegated to small teams. To ensure steady progress, we scheduled regular group writing sessions.

As first drafts of individual sections were completed, they were traded between volunteer teams for feedback, which was then acted on by the original authors. Once all the sections were ready, two of us (BCT and KLM) merged the text into a complete final draft, editing for consistency of language and balance of detail across topics. The draft handbook was subsequently shared with the entire group and discussed in detail over several lab meetings. We further sought expert advice on topics such as working hours, mental health, and equity, diversity and inclusion from departmental working groups and HR staff. Feedback from this second round of discussions was incorporated into the final document, producing the handbook we have today. Overall, the writing process consisted of several bursts of intensive work, interleaved with breaks that allowed us to return to the handbook with fresh eyes.

The final WIN Physics lab handbook is split into three main sections: (i) the roles and expectations of different lab members (including PIs, early-career researchers and doctoral students); (ii) the group culture we aspire to create (including workplace conduct, member wellbeing, good lab citizenship and equity, diversity and inclusion); (iii) our commitment to the development of lab members as researchers (including career development, best research practices, collaboration, travelling and public engagement).

The aim of writing the lab handbook in this way was to create an open forum to discuss our lab ethos and how we aim to work together to create a culture that reflects our values. This conversation was initiated by a PI (KLM), but rapidly evolved into discussions between individual members and the group as a whole. Dialogue was supportive and consensus was routinely reached, with discussion mostly focused on what topics should be included to prevent the handbook becoming burdensomely long.

In several cases, the writing process changed PIs’ thinking. For example, it became clear during the process that one PI, intending to communicate the importance of a healthy work-life balance, would frame their expectations as “I don’t mind what hours you work as long as you are making progress”. However, it became clear during discussion that this statement was interpreted in different ways by different members of the lab: was it intended to support work-life balance, or to indirectly communicate that members of the lab are expected to work long hours? As a result, we now adopt more direct language about working hours and work-life balance, stating that we do not encourage long working hours, and asking lab members to respect the working hours of colleagues.

Similarly, the writing process raised several topics that may be commonly overlooked without a breadth of input. For example, conferences may be held in a country where some group members do not feel travel is morally justifiable or where they personally feel unsafe. Our handbook now explicitly states that members should not feel pressured to travel, and to discuss mitigation options with their supervisor to ensure they do not miss out on career development opportunities.

While it is too early to describe the impact that our lab handbook has had on the WIN Physics group, the early signs are promising. The handbook has been well received by recent new starters, with one saying that they were surprised to see an explicit statement that long working hours are not expected.

Based on our experience, we would recommend writing the handbook over the course of a single term or semester, and the resources described in Box 1 should help with this. Were we to repeat the process, we would lay the foundations of the handbook at a group ’away day’, rather than having a number of short meetings over a period of several weeks.

Contrary to the traditional single-PI lab, the WIN Physics group is a collaboration between three senior and four junior PIs, alongside five senior staff physicists. Our interactions tend to be as a collective, including shared space, frequent co-supervision, and weekly lab meetings of the entire group. While each PI could have authored their own handbook, we opted for a joint document, since our group members identify more strongly with the collective than their own supervisor and we have a strong sense of a shared group culture. The one downside of this joint approach is that the handbook is less specific about a number of topics where the PIs favoured different approaches (e.g., communication style or supervisory preferences). Of course, this would not happen in a lab with just one PI.

## Handbooks to the people!

We are now beginning to promote lab handbooks more broadly across Oxford University. We initially established a series of writing and discussion sessions to facilitate this process, but found that most groups were content to work on their handbooks independently using our resources as a guide. Based on this we have decided to make these resources publicly available (Box 1).

Some colleagues have asked if each handbook needs to be “unique”. Whilst a lab’s handbook explicitly describes the culture and practices of that lab, we recommend striking a balance between writing your “own” lab handbook, harvesting good text from other handbooks to avoid reinventing the wheel, and using standardised text to describe institutional policies. It is also important that a lab handbook is consistent with and refers to other policies and documents in the host department and institution (and also with relevant legislation).

We believe that a lab handbook is an essential resource for any lab, enabling it to operate with a healthy culture and to promote wellbeing amongst group members. Making lab handbooks a common feature of research groups will, we believe, will help to increase job satisfaction, improve lab productivity, and contribute more broadly to positive research culture.

## Data Availability

The following resources are available at https://doi.org/10.5281/zenodo.7419210 (Tendler et al., 2022): (i) an abridged version of the lab handbook written by the Physics group at the Wellcome Centre for Integrative Neuroimaging (WIN); (ii) a video summarising the content of this article that can be used to initiate discussions with group members; (iii) a template containing a series of questions, the answers to which can be used to form the skeleton of a new handbook.
